# Toxin gene profiles, genetic diversity, antimicrobial resistance, and coagulase type of *Staphylococcus aureus* from cream‐filled bakery products

**DOI:** 10.1002/fsn3.1011

**Published:** 2019-03-28

**Authors:** Yong Sun Cho, Myung Ki Lee, Sun Hye Hwang

**Affiliations:** ^1^ Food Analysis Center Korea Food Research Institute Jeollabuk-do Korea; ^2^ Research Group of Traditional Food Korea Food Research Institute Jeollabuk-do Korea

**Keywords:** antibiotic resistance, coagulase serotype, cream‐filled bakery products, *Staphylococcus aureus*, toxin gene

## Abstract

We determined the toxin gene profile, toxin production, antibiotic resistance coagulase serotype, and genetic diversity of 42 coagulase‐positive *Staphylococcus aureus* (CPS) isolates collected from 1,464 cream‐filled bakery products in Korea. Among the CPS isolates, 37 (88.1%) produced enterotoxin genes in combination with another toxin; 26 (61.9%) of the strains were positive for *sea*, 1 (2.4%) for *sea‐seb*, and 4 (9.5%) for *sea‐sec*. Among the strains showing antibiotic resistance, 28 (66.7%) showed resistance to only one antibiotic, whereas nine (21.4%) showed resistance to multiple antibiotics: 4 (9.5%) strains were both *mec*A‐positive and oxacillin‐resistant. Most strains are resistant to at least one antibiotic—benzyl penicillin. The CPS isolates were classified into eight coagulase serotypes. This information will be valuable for assessing the capability risks of CPS food poisoning, contributing a better known of the epidemiology result associated with CPS contamination in bakery products.

## 1 INTRODUCTION

Coagulase‐positive *Staphylococcus aureus* (CPS) is a common pathogen associated with serious community‐related and hospital‐acquired diseases, and has long been considered a major public health problem (Pesavento, Ducci, Comodo, & Nostro, 2007). Bakery products are classified based on their water activity (aw) value; cream‐filled cakes, one of the most common bakery products consumed worldwide, have an intermediate moisture content with an aw value ranging from 0.6 to 0.85 (Smith & Simpson, 1995). Since most bacteria require a high aw for growth, bacterial problems are limited to bakery products with a relatively high moisture content; thus, moisture‐filled bakery products have been implicated in outbreaks of foodborne illness, thereby posing safety concerns (Smith, Daifas, El‐Khoury, Koukuotsis, & El‐Khoury, 2004). In particular, staphylococcal food poisoning (SFP) is a type of intoxication that results from the consumption of foods containing sufficient amounts of one (or more) preformed enterotoxins. Symptoms of SFP include a rapid onset (2–8 hr), with nausea, violent vomiting, and abdominal cramping with or without diarrhea (Argudín, Mendoza, & Rodicio, 2010).

Many of the known *S. aureus* virulence factors can be described as toxins, which are generally defined as poisonous substances. Thus, the distinction from other virulence factors, that is, molecules that increase the potential of a pathogen to cause disease in a broader sense, is that toxins are secreted by the producing organism and interfere directly with the host (Otto, 2014). *S. aureus *is a major human pathogen bacteria that produces various types of toxic proteins such as enterotoxins, toxic shock syndrome toxin 1 (TSST‐1), and exfoliative toxin (ET) all of which can cause food poisoning (Balaban & Rasooly, 2000; Jung et al., 2005). Several types of staphylococcal enterotoxin (SE) have been identified by serological, biochemical analyzes, and molecular genetic diversity over the years, including SEA, SEB, SEC, and SED (Sharma, Rees, & Dodd, 2000). *S. aureus* infection results in different symptoms, which largely depend on the type of toxin secreted; therefore, it is important to be able to reliably and accurately distinguish the type of toxins produced from strains isolated from food to ensure proper treatment and monitor the trends of foodborne illness.

Staphylocoagulase (SC) is an extracellular protein produced by *S. aureus* and has been regarded as a hallmark for the identification of *S. aureus*, arguably the most virulent and clinically important pathogenic species. SC can be classified into eight types of antigenic coagulases (I–VIII). Serotyping methods were developed to identify SC types based on the inhibition of clotting activity with type‐specific antisera and have been widely used for epidemiological studies of *S. aureus*, especially in Japan. Coagulase types VII accounting for about 70% of all outbreaks, III (12%), II (11%), and VI (3%) are, respectively, the first, second, third, and fourth most predominant types of coagulases responsible for food poisoning incidents throughout Japan (Oda, 1998; Shimizu et al., 2000).

*Staphylococcus aureus* is also a frequent cause of human infections, and strains that show antimicrobial resistance, particularly multidrug resistance especially methicillin and vancomycin, have become a major global public health problem in recent. The excessive and inappropriate use of antimicrobial agents is responsible for the emergence and rapid spread of resistant bacterial strains (Kunin, 1993; Fallah, Saei‐Dehkordi, Rahnama, Tahmasby, & Mahzounieh, 2012). In particular, methicillin‐resistant *S. aureus* (MRSA) has increasingly been reported to dominate *S. aureus* infections and is rising in prevalence, becoming a cause of great clinical concern worldwide. The resistant phenotype of MRSA strains is related to the acquisition of a set of genes that are induced and expressed during of β‐lactam exposure; all MRSA strains harbor the *mec*A gene, encoding penicillin‐binding protein 2a (PBP2a) as the key resistance enzyme for virtually all β‐lactam antibiotics (Ảbrók, Lázár, Szécsényi, Deák, & Urbán, 2018; Zhan & Zhu, 2018). Accordingly, polymerase chain reaction (PCR)‐based detection of the highly conserved *mecA *gene is the gold standard for the determination of methicillin resistance in staphylococci. Moreover, *Mup* (encoding pseudomonic acid A), a topical antimicrobial agent produced by *Pseudomonas fluorescens*, has been used to eradicate MRSA from the nasal carriage and staphylococci catheter colonization (Sareyyüpoǧlu, Ozyurt, Haznedaroǧlu, & Ardiç, 2008). An alternative strain‐typing method, which is available commercially, is the DiversiLab typing system (DL; biomérieux, Inc., Durham, NC, USA), which uses the presence of DNA repetitive elements in the organism's genome to determine the genetic relatedness of bacterial and fungal isolates. Cream products more prone to have problems of food poisoning associated with *S. aurues*. However, little studies have been conducted on the characteristics of *S. aureus* isolated from cream‐filled bakery products. The aim of the present study was to characterize different *S. aureus* isolates collected from cream‐filled bakeries in Korea. This characterization was based on the ability of the isolates to produce and express SEs. We further determined the antibiotic susceptibility profiles of the isolates to monitor the presence and prevalence of MRSA strains in cream‐filled bakery products, and the presence of other virulence factors. Furthermore, the relationship between MRSA and their ability to produce virulence factors was evaluated.

## 2 MATERIAL AND METHOD

### 2.1 Bacterial isolates and identification

Forty‐two *S. aureus* strains were isolated from 1,464 samples cream‐filled bakery collected in bakery markets in Korea. In brief, each 25 g sample was enriched with 225 ml of tryptic soy broth with 10% NaCl (Merck, Darmstadt, Germany) at 35°C for 24 hr and then streaked on 10‐μl baird‐parker agar plates (Merck). The typical five colony was conducted coagulase test on baired parker RPF agar (bioMérieux, Marcy L'Etoile, France). Incubation at 35°C 24 hr, a typical colony was selected on blood agar, which was analyzed by a catalase test, gram staining, and biochemical test with the Vitek 2 (bioMérieux, Marcy L'Etoile, France) for identification.

### 2.2 Detection of SEs toxin by the reverse passive latex agglutination (RPLA) assay

Positive *S. aureus* strains were analyzed with a SEs detection kit using RPLA (SET‐RPLA; Denka Seiken, Tokyo, Japan) according to the manufacturer's instructions.

### 2.3 Detection of *Staphylococcus aureus* toxigenic genes by PCR

Chromosome DNA was extracted with an ultraclean™ microbial DNA isolation kit (MO Bio Laboratories Inc., CA, USA) according to the manufacturer's instructions. The samples were then subjected to gradient PCR (Biometra, Goettingen, Germany) with primer pairs listed in Table 1 (Bioneer, Cheongwon, Chungbuk, Korea). PCR was performed AccuPower PCR PreMix (Bioneer, Daejon, Korea) containing 35 ng/μg template DNA and 10 pmol of primer in total 20‐μl reaction mixture. PCR was performed 35 cycles of 95°C for 60 s, 55°C for 60 s, and 72°C for 60 s, which was preceded by a 300 s denaturation step at 95°C and 300 s extension step at 72°C. PCR products were separated by electrophoresis on a 2% seakem agarose gel (Takara Bio, Otsu, Japan) with ethidium bromide solution (1 μg/ml) at 100 V for 30 min and visualized under UV transilluminator.

**Table 1:** Oligonucleotide primers used of staphylococcal toxin genes

Target	Oligonucleotide sequence	Amplicon size (bp)	References
*sea*	TTGGAAACGGTTAAAACGAA	127	Johnson et al. (1991)
	GAACCTTCCCATCAAAAACA		
*seb*	TCGCATCAAACTGACAAACG	478	Johnson et al. (1991)
	GCAGGTACTCTATAAGTGCCTGC		
*sec*	CTCAAGAACTAGACATAAAAGCTAGG	257	Johnson et al. (1991)
	TCAAATCGGATTAACATTATCC		
*sed*	CTAGTTTGGTAATATCTCCTTTAAACG	317	Johnson et al. (1991)
	TTAATACTATATCTTATAGGGTAAACATC		
*mupA*	TATATTATGCGATGGAAGGTTGG	456	Anthony, Connor, Power, and French (1999)
	AATAAAATCCCACATTGTTTCGGTCTAA		
*mecA*	GTAGAAATGACTGAACGTCCGATAA	310	Pérez‐Roth, Claverie‐Martin, Villar, and Mendez‐Alvarez (2001)
	CCAATTCCACATTGTTCGTCTAA		

### 2.4 Antibiotic susceptibility testing

Antibiotic test was performed with the Vitek 2 (bioMérieux, Marcy L'Etoile, France) according to the manufacturer's instructions. The AST‐P601 *S. aureus *card (bioMérieux, Marcy L'Etoile, France) used for contained benzyl penicillin, cefoxitin screen, ciprofloxacin, clindamycin, erythromycin, gentamicin, inducible clindamycin resistance, linezolid, mupirocin, nitrofurantoin, oxacillin, quinupristin/dalfopristin, rifampicin, teicoplanin, telithromycin, tetracycline, trimethoprim/sulfamethoxazole, tigecycline, and vancomycin. The result was interpreted according to the guidelines of the Clinical and Laboratory Standards Institute (CLSI, 2011). Strains *S. aureus* ATCC 29213 and *Enterococcus faecalis *ATCC 29212 were included as quality control strains in the antibiotic susceptibility testing (AST) test.

### 2.5 Identification of MRSA

*Staphylococcus aureus *42 strains were resistant to oxacillin, and then PCR was used to confirm that these strains also harbored the *mecA* and *mupA *gene for identification of MRSA strains (Table 1). PCR was performed 10 cycles of amplification (94°C for 30 s, 64°C for 30 s, and 72°C for 45 s) and 25 cycles of amplification (94°C for 45 s, 50°C for 45 s, and 72°C for 1 min), which was preceded by a 5 min denaturation step at 94°C and followed by a 10 min extension step at 72°C. Each PCR was conducted in triplicate, and the amplified products were separated by electrophoresis as described above.

### 2.6 Identification of coagulase serotype by PCR

Serological coagulase typing was performed using PCR (Table 2) essentially as described above with the following cycling parameters: PCR was performed over 30 cycles of 94°C for 30 s, 52°C for 30 s, and 72°C for 1 min, which was preceded by a 5 min denaturation step at 95°C and followed by a 7 min extension step at 72°C. Each PCR was conducted in triplicate, and the amplified products were separated by electrophoresis as described above.

**Table 2:** Oligonucleotide primers used for detection of coagulase serotype

SC type	Primer name	Oligonucleotide sequence	Amplicon size (bp)	Reference
I	coa1F	GCATTGGATATTTTAGAGAC	644	Sakai et al. (2008)
	coa1R	TCAAAACCTTCACTGTGATT		
II	coa2F	AGAGGCACAATTTACTGGA	342	
	coa2R	CCATCTTTATCAAACTGC		
III	coa3F	GCTCTATATTATTTGGAAGACT	310	
	coa3R	GAAAATCATCCAGTGCTCTC		
IV	coa4F	AAAGTGAAAATCCACATTCTAG	490	
	coa4R	TCTCTATTTTCAGGCTTATTA		
V	coa5F	GAGAAAGATATTTAAAAGCTGG	482	
	coa5R	TTCTTTGTTATCTTTAGGGCT		
VI	coa6F	TTACTTTTGGGGGAAAATCG	269	
	coa6R	CCATAGTTAGATTATATACAC		
VII	coa7F	TTCATTTACTGGATCAGC	217	
	coa7R	GTTAAATCGCCAAGATCG		
VIII	coa8F	CACTTATTACTGGGGAGT	358	
	coa8R	CTTTTTCGACTGTATATCATC		

### 2.7 Rep‐PCR (DiversiLab) genetic typing system

*Staphylococcus aureus *strains were cultured on blood agar for 24 hr at 35°C. Genomic DNA was extracted as described above, and the DNA was diluted of 35 ng/μg. Rep‐PCR was performed using the DiversiLab *Staphylococcus* kit (Bacterial BarCodes, Inc., Houston, TX, USA) accordance with the manufacturer's product insert. PCR was performed on a gradient using the following parameters: 94°C for 2 min and then 35 cycles of amplification (94°C for 30 s, 45°C for 30 s, and 70°C for 90 s), with 70°C for 3 min. Analysis of rep‐PCR products was implemented using the DiversiLab((bioMérieux, Marcy L'Etoile, France) in which the amplified fragments detected using a microfluidics Labchip with the Agilent 2100 Bioanalyzer (Agilent Technologies, Palo Alto, CA, USA). The DNA fingerprint patterns were automatically downloaded onto a secure laboratory‐designated DiversiLab program. Agreement between methods was assessed at different rep‐PCR SI cutoffs, including 80%, 85%, and 90%, as generated by the DiversiLab software, and the relatedness was determined by cluster analysis according to the guidelines provided by the manufacturer.

## 3 RESULT AND DISCUSSION

### 3.1 SEs production

Coagulase‐positive *S. aureus *strains are readily killed by pasteurization or cooking; however, the microorganism's enterotoxins tend to be heat‐stable and can survive even at the high temperatures (121°C) used to process low‐acid canned foods (Stewart, Cole, & Schaffner, 2003). Among the 42 CPS strains isolated from cream‐filled bakery products, RPLA analysis showed that a total of 10 strains (23.8%) produced one or more SEs: 19.0%, 2.4%, and 2.4% of the strains produced SEA, SEA + SEB, and SEA + SEC, respectively (Table 3). In RPLA, the most commonly employed and commercially available enterotoxin identification method, the enterotoxins are identified by specific antibodies (Sharma et al., 2000). For further confirmation, we also amplified the enterotoxin‐encoding genes in the CPS strains; *sea* was the predominant gene, detected in 26 strains (61.9%, Table 3). Overall, 11.9% (5/42) of the *S. aureus* strains harbored the targeted classical SEs genes (*sea, seb*, and *sec*), including *sea‐sec* (9.5%, 4/42) and *sea‐seb* (2.4%, 1/42). Complete agreement of the results between the RPLA and PCR identification methods was obtained for nine of the 42 strains (21.4%). This level is much lower than the agreement rate of 93.9% reported by Kerouanton et al. (2007). Therefore, it is necessary to consider both the phenotype and genotype to best distinguish the enterotoxin of *S. aureus*.

**Table 3:** Phenotype and genotype of enterotoxin CPS identified

Phenotype of enterotoxin (%)					
SEA	SEB	SEC	SED	SEA + SEB	SEA + SEC
8 (19.0)	1 (2.4)	0 (0.0)	0 (0.0)	0 (0.0)	1 (2.4)

Genotype of enterotoxin (%)					
*sea*	*seb*	*sec*	*sed*	*sea + seb*	*sea + sec*
26 (61.9)	0 (0.0)	0 (0.0)	0 (0.0)	1 (2.4)	4 (9.5)

In Korea, SEA is the most frequent SE type; however, other SEs, including novel SEs, have previously been reported to be detected together with SEA (Cha et al., 2006; Hyeon et al., 2013). The SEA toxin type has also been associated with several *S. aureus *outbreaks linked to bakery products (Stewart et al., 2003). However, various toxins have been detected in such outbreaks, and the major toxin type varies widely in different countries. Therefore, SE identification could provide information on the origin of the foodborne pathogen, and their genetic characterization and geographical characterization. But new pathogenic factors are being reported and so further studies on novel clones with unknown genetic backgrounds and their relation are needed (Kerouanton et al., 2007), including specific investigations for different types of foods and countries.

Foodborne outbreaks associated with cream‐filled bakery products are frequently attributed to inadequate refrigeration during manufacturing or storage (Bryan, 1976). Therefore, there is a need to carefully manage the production of such products from the manufacturing stage to the sales and distribution stage up to consumption.

### 3.2 AST and MRSA identification

Antimicrobial resistance analysis showed that 37 of the 42 strains were resistant to more than one antibiotic tested. The highest resistance rate was found to P (88.1%), followed by OX (9.5%), GM (4.8%), TE (4.8%), and E (2.4%). There was no antibiotic resistance to VA detected in any strain (Table 4).

**Table 4:** Antimicrobial resistance of *Staphylococcus aureus* strains isolated from cream‐filled bakery products

Antimicrobial agent	Resistance (%)	Intermediate (%)	Susceptible (%)
P	88.1	0	11.9
O X	9.5	0	90.5
G M	4.8	9.5	85.7
CIP	0	0	100
E	2.4	2.4	95.2
TEL	0	0	100
CM	0	0	100
QDA	0	0	100
LNZ	0	0	100
TEC	0	0	100
VA	0	0	100
TE	4.8	0	95.2
TGC	0	0	100
FT	0	0	100
MUP	0	0	100
RA	0	0	100
SXT	0	0	100

Note

CIP: ciprofloxacin; CM: clindamycin; E: erythromycin; FT: nitrofurantoin; GM: gentamicin; LNZ: linezolid; MUP: mupirocin; OX: oxacillin; P: benzyl penicillin; QDA: quinupristin/dalfopristin; RA: rifampicin; SXT: trimethoprim/sulfamethoxazole; TE: tetracycline; TEC: teicoplanin; TEL: telithromycin; TGC: tigecycline; VA: vancomycin.

Overall, nine (21.4%) multidrug‐resistant strains were detected. In particular, the OX‐resistant MRSA strains were resistant to three or five antibiotics (Table 5). PCR confirmation and the PBP2' RPLAS reaction of these candidate MRSA strains identified six strains (14.2%) with the *mec*A gene. One strain harbored the *mec*A and *mup*A genes simultaneously. However, four strains were OX‐ and multi‐resistant strains. Two strains were susceptible to both OX and OXSF antibiotics (Table 6).

**Table 5:** Multiple drug resistance of *Staphylococcus aureus* isolates from cream‐filled bakery products

No. of drugs	Resistant patterns					No. of resistant strains	Total (%)
5	P	OXSF	OX	E	ICR	1	1 (2.4)
3	P	OXSF	OX			3	3 (7.1)
2	P	TE				2	5 (11.9)
	P	GM				2	
	P	ICR				1	
1	P					28	28 (66.7)
0						5	5 (11.9)

Note

E: erythromycin; ICR: inducible clindamycin resistance; OX: oxacillin; OXSF: cefoxitin screen; P: benzyl penicillin; TE: tetracycline.

**Table 6:** List of MRSA strains isolated from cream‐filled bakery products

Sample ID	*mec* A		mup A	OXSF	P	OX	ICR	E
	PCR	PBP2'						
13‐112	+	+	−	+	R	R	+	R
13‐115	+	+	−	+	R	R	−	S
13‐141	+	+	+	−	R	S	−	S
13‐222	+	+	−	+	R	R	−	S
13‐224	+	+	−	−	R	S	−	S
13‐244	+	+	−	+	R	R	−	S

Note

R: resistance; S: susceptible.

In accordance with the CLSI guidelines, the *mec*A‐positive and OX‐resistant strains were concluded to be MRSA. Four strains were found to be positive for *mec*A and were consistently resistant to OX. All MRSA strains were resistant to all β‐lactams tested, including penicillin. Regarding the involvement of MRSA in food poisoning, Jones, Kellum, Porter, Bell, and Schaffner (2002) reported the first outbreak of gastrointestinal illness caused by community‐acquired MRSA. Food is also an important source for the transfer of antimicrobial resistance, which can occur by means of residues of antibiotics, through the transfer of resistant foodborne pathogens, or through the ingestion of resistant strains of the original food microflora and ultimate resistance transfer to pathogenic microorganisms (Hennekinne, Buyser, & Dragacci, 2012). Therefore, studies on the antimicrobial resistance of microorganisms isolated from foods are very important and can be used as basic data to prevent community infection. Moreover, further study is necessary to test the hypothesis that MRSA can be cross‐contaminated between humans and foods, emphasizing the importance of improving hygiene in food production practices as a countermeasure to limit the spread of antimicrobial‐resistant organisms and foods.

### 3.3 Identification of coagulase serotype by PCR

Serological coagulase typing was performed using the PCR *coa* genes of eight different serotypes (Table 7). The serological coagulase types of the 42 isolates were classified into seven categories according to the toxin genes: coagulase type V strains 47.6% (*n = *20) encoded *sea *(*n* = 12) or *sea‐sec *(*n* = 2), type IV 14.3% (*n* = 6) encoded *sea *(*n* = 5), and type VII 14.3% (*n = *6) encoded *sea *(*n* = 3)*, sea + seb *(*n* = 1), and *sea + sec *(*n* = 1). Coagulase type VII strains have been the predominant types responsible for food poisoning since the 1980s. Coagulase VII, which accounted for about 75.6% of the total number of outbreaks, was the predominant type, and the second most prevalent type was coagulase IV (Cha et al., 2006). Coagulase types VII (70%), III (12%), II (11%), and VI (3%) are most predominant types of coagulases responsible for food poisoning incidents throughout Japan (Shimizu et al., 2000). Another report showed that the four predominant coagulase genotypes of *S. aureus* (I, II, VII, and VIII) were more common than isolates harboring the six rare coagulase types (III, IV, V, VI, IX, and X (Moon et al., 2007). In the present study, the V type was the most dominant, although VII and IV, which are genotypes related to food poisoning problems, were also isolated. These results differ from the common coagulase type of *S. aureus *known to cause food poisoning, but warrant further study. In addition, it is necessary to further examine the characteristics of isolated *S. aureus* and relate these characteristics to determine the causes of food poisoning.

**Table 7:** Coagulase types of *Staphylococcus aureus *strains identified by PCR

Coagulase serotype	I	II	III	IV	V	VI	VII	VIII	N*
Strains (%)	0 (0)	3 (7.1)	1 (4.8)	6 (14.3)	20 (47.6)	2 (4.7)	6 (14.3)	1 (2.4)	3 (7.1)

Note

N*: not detected.

### 3.4 Test method: rep‐PCR DiversiLab microbial typing system

**Figure 1:**
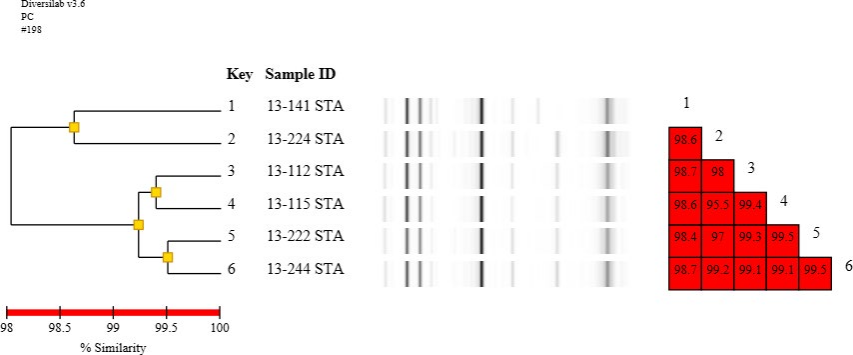
Rep‐PCR profile comparing MRSA isolates collected from cream‐filled bakery products

Typing by DiversiLab revealed that the six MRSA isolates were genetically diverse with a 95% similarity. In particular, the homology of strains was very high (98.6%), and strains resistant to OX showed a homology of 99.9% or more (Figure 1). Moreover, these results confirm that DiversiLab, although considered to be less discriminatory than pulsed‐field gel electrophoresis for typing MRSA, can provide information that is useful for infection control investigations in hospitals (Tenover et al., 2009). Cream‐filled breads are one of the most common bakery products consumed worldwide. However, these products are particularly susceptible to physical, chemical, and microbiological hazards during storage at the outlet, which is exacerbated by the general lack of overview related to food safety (Smith et al., 2004). Food safety are complex issues involving many inter‐related variables parameter, including moisture, aw, pH, preservatives, microbial ecology of the food nutrient matrix, packaging, and storage temperature. Overall, bakery products with a particularly high moisture content have been implicated in several outbreaks of foodborne disease. Therefore, continuing education in food hygiene and safety for bakery personnel, food handlers, and, indeed, the general public is also a critical component in the battle against foodborne disease. In our study, we showed the presence of resistant strains of *S. aureus* in bakery products sold across Korea and identified SEA as the predominant enterotoxin. These results confirmed potential risks of *S. aureus* contamination and useful for will be assessment of the in cream‐filled bakery products: profiling of the toxin phenotype, genotype, antimicrobial resistance, coagulase type, and genetic diversity.

## ETHICAL APPROVAL AND CONSENT TO PARTICIPATE

Ethics approval and consent to participate is not applicable to this manuscript, since it does not report on or involve the use of any animal or human data or tissues.

## CONFLICT OF INTEREST

The authors declare that they do not have any conflict of interest.
